# Compositional and Functional Differences in the Human Gut Microbiome Correlate with Clinical Outcome following Infection with Wild-Type Salmonella enterica Serovar Typhi

**DOI:** 10.1128/mBio.00686-18

**Published:** 2018-05-08

**Authors:** Yan Zhang, Arthur Brady, Cheron Jones, Yang Song, Thomas C. Darton, Claire Jones, Christoph J. Blohmke, Andrew J. Pollard, Laurence S. Magder, Alessio Fasano, Marcelo B. Sztein, Claire M. Fraser

**Affiliations:** a Institute for Genome Sciences, University of Maryland School of Medicine, Baltimore, Maryland, USA; b Oxford Vaccine Group, Department of Paediatrics, University of Oxford, Oxford, United Kingdom; c NIHR Oxford Biomedical Research Centre, Oxford, United Kingdom; d Department of Epidemiology and Preventive Medicine, University of Maryland, Baltimore, Maryland, USA; e Department of Pediatrics, Mucosal Immunology and Biology Research Center, Harvard Medical School, Massachusetts General Hospital, Charlestown, Massachusetts, USA; f Department of Pediatrics, Center for Vaccine Development, University of Maryland School of Medicine, Baltimore, Maryland, USA; g Department of Medicine, University of Maryland School of Medicine, Baltimore, Maryland, USA; h Department of Microbiology and Immunology, University of Maryland School of Medicine, Baltimore, Maryland, USA; Rutgers, The State University of New Jersey

**Keywords:** 16S rRNA gene profiling, *Salmonella*, immunization, metatranscriptomics, methanogens, microbiome, typhoid disease

## Abstract

Insights into disease susceptibility as well as the efficacy of vaccines against typhoid and other enteric pathogens may be informed by better understanding the relationship between the effector immune response and the gut microbiota. In the present study, we characterized the composition (16S rRNA gene profiling) and function (RNA sequencing [RNA-seq]) of the gut microbiota following immunization and subsequent exposure to wild-type Salmonella enterica serovar Typhi in a human challenge model to further investigate the central hypothesis that clinical outcomes may be linked to the gut microbiota. Metatranscriptome analysis of longitudinal stool samples collected from study subjects revealed two stable patterns of gene expression for the human gut microbiota, dominated by transcripts from either *Methanobrevibacter* or a diverse representation of genera in the *Firmicutes* phylum. Immunization with one of two live oral attenuated vaccines against *S.* Typhi had minimal effects on the composition or function of the gut microbiota. It was observed that subjects harboring the methanogen-dominated transcriptome community at baseline displayed a lower risk of developing symptoms of typhoid following challenge with wild-type *S.* Typhi. Furthermore, genes encoding antioxidant proteins, metal homeostasis and transport proteins, and heat shock proteins were expressed at a higher level at baseline or after challenge with *S.* Typhi in subjects who did not develop symptoms of typhoid. These data suggest that functional differences relating to redox potential and ion homeostasis in the gut microbiota may impact clinical outcomes following exposure to wild-type *S.* Typhi.

## IMPORTANCE

*S.* Typhi is a significant cause of systemic febrile morbidity in settings with poor sanitation and limited access to clean water. It has been demonstrated that the human gut microbiota can influence mucosal immune responses, but there is little information available on the impact of the human gut microbiota on clinical outcomes following exposure to enteric pathogens. Here, we describe differences in the composition and function of the gut microbiota in healthy adult volunteers enrolled in a typhoid vaccine trial and report that these differences are associated with host susceptibility to or protection from typhoid after challenge with wild-type *S*. Typhi. Our observations have important implications in interpreting the efficacy of oral attenuated vaccines against enteric pathogens in diverse populations.

## INTRODUCTION

Salmonella enterica serovar Typhi (*S.* Typhi) is a human-restricted enteric pathogen that causes typhoid fever affecting an estimated 11.9 to 26.9 million people annually (1), principally those living in low-resource or conflict settings and travelers to regions of endemicity. Ty21a, a live attenuated oral vaccine, is one of two widely available vaccines for preventing typhoid (2). Immunization with Ty21a induces both humoral immunity (mucosal and serum antibodies and B memory cells) and cell-mediated immune (CMI) responses (3). Local and systemic antibodies to *S.* Typhi antigens are thought to be active against typhoid bacilli when they are extracellular, whereas the CMI response plays an important role in eliminating intracellular *S.* Typhi (4). In a prior study, we investigated the relationship between the gut microbiota and defined *S.* Typhi-specific immunological responses in volunteers receiving the Ty21a vaccine. We observed a significant association between a multiphasic CMI response and a more diverse gut microbiota—with more than 200 operational taxonomic units (OTUs) differentiating multiphasic from late responders (5).

The development of a human challenge model provided us with a unique opportunity to characterize the composition and function of the gut microbiota following immunization and subsequent exposure to *S.* Typhi and to further investigate the central hypothesis that mucosal immune responses are linked to the gut microbiota. Mounting evidence suggests that microbiota composition may be linked to immunogenicity and oral vaccine efficacy (6–9). For example, excessive bacterial growth in the small intestine of children living in resource-limited settings might contribute to the low antibody titers seen in response to an oral live attenuated Vibrio cholerae vaccine, CVD 103-HgR (10). Moreover, reduced vaccine efficacy and immunogenicity in resource-limited regions have also been reported with other vaccines, including rotavirus and oral polio vaccines (8, 11, 12). Despite these limitations, live attenuated oral vaccines continue to have great potential to reduce disease transmission and prevent clinical infection around the world. Through studies such as those presented here, there is the potential for new insights that may ultimately be leveraged to improve protective efficacy of oral vaccines against typhoid and other enteric infections.

## RESULTS

To investigate the efficacy of immunization with two live oral attenuated vaccines against *S.* Typhi (3 doses of Ty21a vaccine or one dose of the candidate vaccine, M01ZH09) and subsequent challenge with wild-type (wt) *S.* Typhi Quailes strain and the effects on the composition and function of the gut microbiota, we carried out longitudinal 16S rRNA gene and metatranscriptomic analyses of stool samples obtained from 30 healthy adult trial subjects who were randomly assigned to one of the three vaccine groups in a study performed in Oxford, United Kingdom (13). These volunteers were healthy males or nonpregnant females, between 18 and 60 years of age and with no history of typhoid vaccination or *S.* Typhi infection.

### Compositional profiling of microbial communities using 16S rRNA gene sequencing.

**FIG 1:**
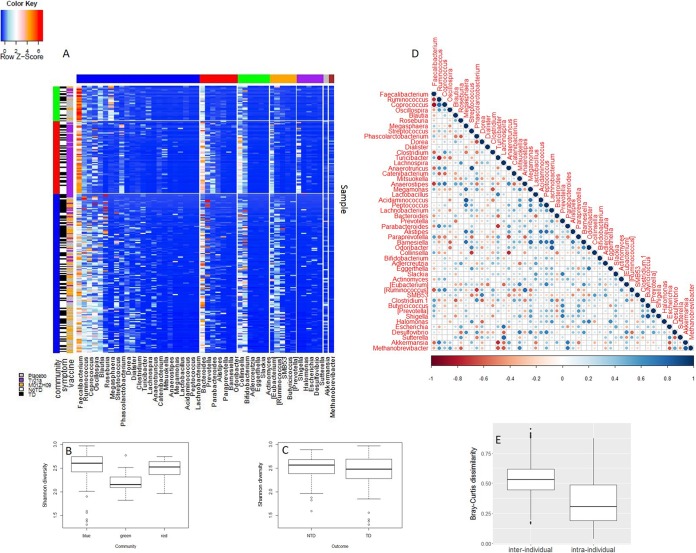
16S rRNA profiling of longitudinal samples. (A) Longitudinal 16S rRNA data were used as input for an in-house *k*-means clustering pipeline. Vertical side bars (green, red, and blue) represent three clusters that were identified. These clusters are dominated by (i) *Faecalibacterium*, (ii) *Faecalibacterium* and *Bacteroides*, and (iii) *Firmicutes*, respectively. Horizontal side bars are color coded as follows: blue, *Firmicutes*; red, *Bacteroidetes*; green, *Actinobacteria*; purple, *Proteobacteria*; gray, *Verrucomicrobia*; brown, *Euryarchaeota*; orange, other. (B and C) Shannon diversity was calculated for each community (B) and clinical outcome (no TD [NTD] or TD) (C). (D) Relationship between genera was assessed by Spearman’s rank correlation coefficient using average genus abundances at baselines. The size and color of circles represent the correlation shown in the color bar. (E) Bray-Curtis dissimilarity values were calculated and compared between different subjects at the same time point (i.e., interindividual) as well as within each individual among different time points (i.e., intraindividual).

We generated 89 million high-quality sequence reads from bacterial 16S rRNA gene V3-V4 amplicons, with a mean (±standard deviation [SD]) of 157,048 (±160,204) reads per stool sample. A *k*-means clustering pipeline grouped the 16S rRNA gene data from all samples into three clusters characterized by (i) high relative abundance of *Faecalibacterium* (Fig. 1A, green vertical bar), (ii) high relative abundance of *Faecalibacterium* and *Bacteroides* (Fig. 1A, red vertical bar), or (iii) a high relative abundance of reads from diverse *Firmicutes* (Fig. 1A, blue vertical bar). The community that was dominated by reads from *Faecalibacterium* (green) displayed a significantly lower taxonomic diversity than the other two communities (linear mixed-effects model, *P* value ≤ 0.01) (Fig. 1B). No significant difference in taxonomic diversity as calculated with the Shannon diversity index was observed between subjects who did (typhoid diagnosis [TD]) or did not (no TD) develop typhoid (Fig. 1C). It was noted that the distribution of samples within the 16S rRNA gene clusters was skewed with regard to vaccine group, with the placebo recipients represented in the *Faecalibacterium*-dominated or diverse *Firmicutes* community, the majority of Ty21a recipients represented in the *Faecalibacterium* and *Bacteroides*-dominated community, and the majority of M01ZH09 recipients represented in the diverse *Firmicutes* community. These differences, reflecting the random assignment of subjects to specific vaccine groups at the time of recruitment, potentially make comparison of outcomes based on immunization challenging.

Spearman’s rank correlation was applied, using averaged genus abundances from two baseline samples, to identify relationships between taxa (Fig. 1D). Within the *Bacteroidetes* phylum, an inverse correlation between *Bacteroides* and *Prevotella* was observed as previously reported (14). *Methanobrevibacter* was positively correlated with *Akkermansia*, *Parabacteroides*, *Ruminococcus*, *Coprococcus*, and *Oscillospira* and inversely correlated with *Faecalibacterium* and *Roseburia*. Consistent with previous results from our laboratory and others, we observed marked interindividual variability in the composition of the microbiota, although longitudinal samples collected from the same individual displayed higher similarity to each other than to those collected from unrelated individuals (Fig. 1E).

### Taxonomic profiling of microbial communities using metatranscriptomic analysis.

mRNA was extracted from a subset of the same stool samples that were processed for 16S rRNA gene profiling, including samples collected at two baselines, two vaccination time points, prechallenge, and two postchallenge time points (day 1 and day 7 after challenge). Whole-community RNA sequencing (RNA-seq) was performed on the Illumina HiSeq 2000 platform, generating an average of 71.6 ± 26.2 million paired-end reads per sample. After quality control (QC) and filtration of rRNA and human RNA reads, 50.4 ± 26.0 million reads per sample were used as input for transcriptomic community profiling using unique clade-specific marker genes by MetaPhlAn v2.0 (15).

**FIG 2:**
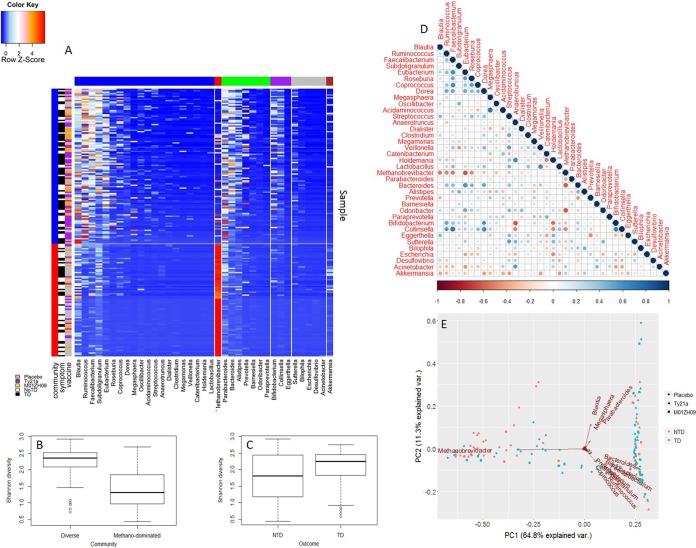
Metatranscriptome profiling of longitudinal samples. (A) Longitudinal metatranscriptome data were used as input for an in-house *k*-means clustering pipeline. Vertical side bars (red and blue) represent two community types that were identified: methanogen-dominated and diverse *Firmicutes*, respectively. Horizontal side bars are color coded as follows: blue, *Firmicutes*; red, *Euryarchaeota*; green, *Bacteroidetes*; purple, *Actinobacteria*; gray, *Proteobacteria*; brown, *Verrucomicrobia*. (B) Shannon diversity values were calculated for the two communities and were significantly different (*P* values ≤ 0.01). (C) Shannon diversity values calculated between the two clinical outcomes (no TD [NTD] or TD) were not significantly different (*P* values = 0.15). (D) Relationship between genera was assessed by Spearman’s rank correlation coefficient using average genus abundances at baselines. Size and color of circles represent the correlation shown in the color bar. (E) Principal-component analysis was applied to visualize dissimilarities among samples.

The *k*-means clustering pipeline was used to analyze the taxonomic composition of the entire metatranscriptomic data set, and this analysis identified two clusters. These were (i) a set of samples in which *Methanobrevibacter* transcripts were highly abundant (59.0% ± 23.4%) and (ii) another set of samples characterized by transcript abundance from a large number of genera across multiple phyla (Fig. 2A), a finding similar to that previously described by our laboratory (16). These findings were supported by principal-component analysis (PCA), which confirmed two main clusters distinguished by the abundance of *Methanobrevibacter* transcripts (Fig. 2E). Using a linear mixed-effects model, the Shannon diversity of the methanogen dominated (1.45 ± 0.60) and the diverse *Firmicutes* (2.25 ± 0.42) cluster was found to be significantly different (*P* values ≤ 0.01) (Fig. 2B). In contrast, the Shannon diversity values of the subjects who did or did not develop typhoid were not significantly different (*P* value = 0.15) (Fig. 2C).

Spearman’s rank correlation coefficient was applied to assess the pairwise relationship between genera in the metatranscriptomic data set using averaged genus abundances from two baseline samples (Fig. 2D). *Methanobrevibacter* showed a strong negative correlation with several *Firmicutes* genera, including *Eubacterium* (ρ = −0.64, *P* value ≤ 0.01), *Faecalibacterium* (ρ = −0.54, *P* value ≤ 0.01), *Ruminococcus* (ρ = −0.51, *P* value ≤ 0.01), *Blautia* (ρ = −0.55, *P* value ≤ 0.01), and *Roseburia* (ρ = −0.49, *P* value = 0.01), as well as *Bacteroides* (ρ = −0.61, *P* value ≤ 0.01), whereas these genera were positively correlated with each other. *Bacteroides* and *Prevotella* also displayed an inverse correlation (ρ = −0.45, *P* value = 0.02), similar to what was observed in the 16S rRNA gene data set. We assessed overall correlation between the 16S rRNA gene data and the metatranscriptome data (see Fig. S1 in the supplemental material); however, no significant relationship was observed, as previously described (16).

10.1128/mBio.00686-18.2 FIG S1  Taxonomic abundance of the human gut microbiome estimated by 16S rRNA and metatranscriptome sequencing. The two heat maps represent the same data presented in Fig. 1 and 2. In the middle panel, lines have been drawn to connect the same samples between the two heat maps. Download FIG S1, PDF file, 0.4 MB. Copyright © 2018 Zhang et al. 2018 Zhang et al. This content is distributed under the terms of the Creative Commons Attribution 4.0 International license.

**FIG 3:**
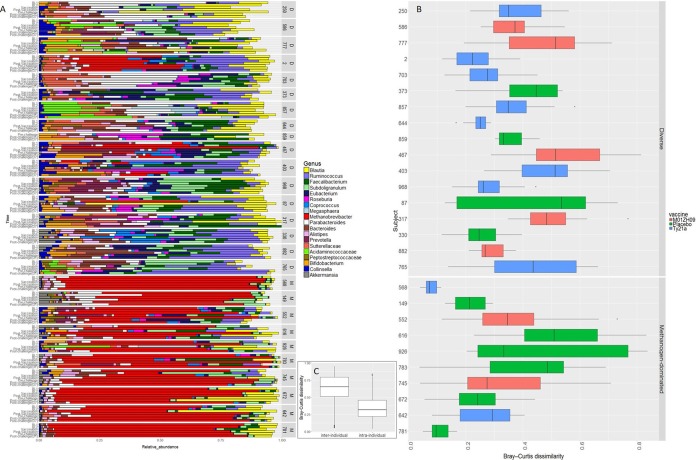
RNA-seq transcript dynamics and dissimilarity across subjects. (A) Relative genus abundance for each subject over time. D and M stand for diverse and *Methanobrevibacter*-dominated community, respectively. (B) Bray-Curtis dissimilarity for each of the subjects in this study. Each box plot represents the dissimilarity in the composition of the microbiota between all longitudinal samples collected from the same subject. Five statistical values are indicated on each box plot, including minimum, first quartile, median, third quartile, and maximum. (C) Bray-Curtis dissimilarity values were calculated and compared among different subjects at the same time point (i.e., interindividual) as well as within each individual among different time points (i.e., intraindividual).

Pairwise comparison of samples within individuals using the Bray-Curtis (B-C) dissimilarity index allowed for quantification of taxonomic changes in the metatranscriptomic data set over time (17). Longitudinal analysis of the metatranscriptomic data revealed that the taxonomic profiles were relatively stable within an individual, regardless of vaccine received or the outcome after challenge (Fig. 3A and B; Table S1). The methanogen-dominated community (B-C dissimilarity = 0.30 ± 0.20) appeared to be more stable over time than the diverse *Firmicutes* community, although this was not statistically significant (B-C dissimilarity = 0.38 ± 0.15; *P* value = 0.096 using linear mixed-effects model) (Fig. 3C).

10.1128/mBio.00686-18.4 TABLE S1  Bray-Curtis dissimilarity for each subject over time. Abbreviations: BL, baseline; Vacc, vaccination; PostVacc, postvaccination; PreChall, prechallenge; Post1D, 1 day postchallenge; Post7D, 7 day postchallenge. Download TABLE S1, PDF file, 0.2 MB. Copyright © 2018 Zhang et al. 2018 Zhang et al. This content is distributed under the terms of the Creative Commons Attribution 4.0 International license.

### Functional differences between transcriptome community types.

**FIG 4:**
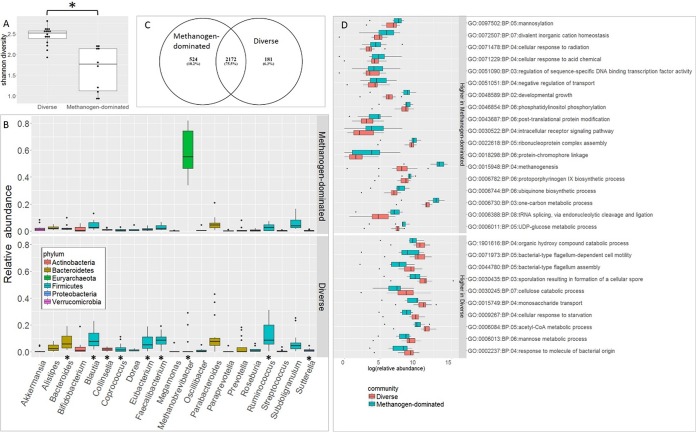
Comparative analysis of RNA-seq data at baseline. (A) Shannon diversities of the two transcriptome communities were significantly different (*P* < 0.001). (B) Relative abundances of genera in each transcriptome community. Genera with significantly different abundances between the two communities are denoted with asterisks. (C) Number of unique and common KEGG orthologues detected in each transcriptome community. (D) Gene ontology groups that were significantly different between the two transcriptome communities are shown (false discovery rate, ≤0.05).

Comparison of the functional properties of the transcriptome communities as represented by Gene Ontology (GO) terms revealed significant and consistent differences over time (Fig. 4). A total of 2,877 KEGG orthologs (KOs) were identified in the metatranscriptomic data set with 75.5% of these shared between the methanogen-dominated and diverse communities. Each community type also contained a subset of unique KOs representing 18.2% of the total in the methanogen-dominated community versus 6.3% in the diverse *Firmicutes* community. As represented by GO terms, genes present only in the methanogen-dominated community were involved in methanogenesis (GO:0015948), cellular response to radiation (GO:0071478), divalent inorganic cation homeostasis (GO:0072507), protoporphyrinogen IX biosynthesis (GO:0006782), one-carbon metabolism (GO:0006730), and posttranslational protein modification (GO:0043687). In contrast, the diverse *Firmicutes* community was characterized by a significantly greater abundance of genes involved in flagellum assembly (GO:0044780), flagellum-dependent cell motility (GO:0071973), sporulation resulting in formation of a cellular spore (GO:0030435), monosaccharide transport (GO:0015749), mannose metabolism (GO:0006013), and cellulose catabolism (GO:0030245). We evaluated the quality of the RNA-seq results by quantitative reverse transcriptase PCR (qRT-PCR), using a subset of genes that were differentially expressed between the two transcriptome communities (flagellar hook protein FlgE [KO02390], flagellin [KO02406], an uncharacterized protein from *Methanobrevibacter* [KO06933], and methanol corrinoid protein [KO14081]) (Table S2). We observed a high correlation of fold changes in these genes as measured by both techniques (Fig. S1), lending additional support to the notion that these transcriptome communities differ with regard to function.

10.1128/mBio.00686-18.5 TABLE S2  Primers used in this study. Download TABLE S2, PDF file, 0.3 MB. Copyright © 2018 Zhang et al. 2018 Zhang et al. This content is distributed under the terms of the Creative Commons Attribution 4.0 International license.

### Global differential gene expression associated with vaccination.

There was no change in the global gene expression profile of the gut microbiome (i.e., the sum of the gene expression profile from all taxa), before and after placebo treatment, as expected. Similarly, relatively few genes were differentially expressed before and after immunization in volunteers who received Ty21a (4 genes downregulated and 4 genes upregulated following immunization). These findings are consistent with a previous report that immunization with Ty21a has minimal effects on the gut microbiota (16). In contrast, 72 genes were differentially expressed globally in volunteers receiving the M01ZH09 vaccine. Twenty-nine methanogenesis-associated genes were upregulated in this treatment group. While it is possible that this difference may be related to vaccination, it is perhaps more likely that the differentially expressed (DE) genes reflect fluctuation in the taxonomic composition in these samples over time.

### Association between community type and clinical outcome.

Of the 30 study subjects included in this study, 13 individuals (43%) did not fulfill the definition for typhoid diagnosis (TD) after challenge with wild-type *S.* Typhi, including 50% of the placebo control group (13). The availability of longitudinal 16S rRNA gene and metatranscriptome data from study participants provided the opportunity to identify potential differences in the composition and/or function of the gut microbiota that was associated with clinical outcome.

We observed a difference in clinical outcome (TD versus no TD) following challenge with wild-type *S.* Typhi that was associated with transcriptome communities. Sixty percent of the volunteers whose microbiome contained a high relative abundance of *Methanobrevibacter* transcripts did not develop TD symptoms following challenge. In contrast, 65% of the volunteers whose microbiome contained few, if any, *Methanobrevibacter* transcripts did exhibit clinical symptoms consistent with TD. To assess whether clinical outcome was distributed uniformly across each gut transcriptome community (methanogen-dominated or diverse *Firmicutes*), the chi-square test was applied (*P* value of 0.096), and the results, albeit not statistically significant, suggested that a difference between the gut communities was associated with a risk of TD.

A mixed-effects regression model was applied to the longitudinal data to estimate the mean relative abundance of each taxon at different time points (baseline, postvaccine, and postchallenge) among those subjects who did or did not develop TD, and *P* values were computed to compare the two groups at each time point (Table 1). This analysis revealed a significant association between the high abundance of *Methanobrevibacter* transcripts and lack of typhoid disease following challenge. In contrast, *Eubacterium* transcripts were found at a significantly higher relative abundance in subjects who developed TD at baseline, postvaccine, and postchallenge, suggesting a relationship between *Eubacterium* and TD; *Faecalibacterium*, *Bacteroides*, *Alistipes*, and *Prevotella* also showed significantly higher relative abundances in TD subjects during one or two of these time periods.

**TABLE 1:** Mean relative abundance of genera at different time points^a^

Genus	Baseline		Postvaccine		Postchallenge	
	No TD	TD	No TD	TD	No TD	TD
*Blautia*	0.069	0.071	0.084	0.127	0.066	0.079
*Ruminococcus*	0.090	0.081	0.088	0.074	0.094	0.100
***Faecalibacterium***	0.048	0.082	0.048	0.077	0.045^1^	0.087^1^
*Subdoligranulum*	0.075	0.052	0.055	0.051	0.048	0.055
***Eubacterium***	0.028^2^	0.069^2^	0.030^3^	0.061^3^	0.035^4^	0.073^4^
*Roseburia*	0.014	0.015	0.040	0.018	0.015	0.026
*Coprococcus*	0.021	0.016	0.019	0.015	0.018	0.021
*Dorea*	0.009	0.011	0.007	0.012	0.010	0.015
***Methanobrevibacter***	0.315	0.180	0.346	0.170	0.367^5^	0.142^5^
*Parabacteroides*	0.071	0.114	0.065	0.075	0.050	0.080
***Bacteroides***	0.052	0056	0.028^6^	0.063^6^	0.034	0.059
***Alistipes***	0.025	0.029	0.018	0.025	0.021^7^	0.038^7^
***Prevotella***	0.010	0.037	0.006^8^	0.040^8^	0.019	0.030
***Sutterellaceae*, unknown genus**	0.020^9^	0.048^9^	0.025	0.050	0.016^10^	0.035^10^
*Bifidobacterium*	0.017	0.029	0.012	0.028	0.043	0.050
*Collinsella*	0.011	0.017	0.013	0.016	0.020	0.018
*Sutterella*	0.006	0.011	0.007	0.013	0.006	0.014
*Akkermansia*	0.008	0.017	0.010	0.009	0.008	0.010

a Pairs with a *P* value of ≤0.1 are denoted by superscript numbers, and the corresponding genera are marked in bold. Superscript numbers correspond to *P* values as follows: 1, *P* = 0.075; 2, *P* = 0.033; 3, *P* = 0.097; 4, *P* = 0.057; 5, *P* = 0.058; 6, *P* = 0.061; 7, *P* = 0.021; 8, *P* = 0.078; 9, *P* = 0.071; 10, *P* = 0.091.

### Taxon-specific differential gene expression associated with clinical outcome.

**FIG 5:**
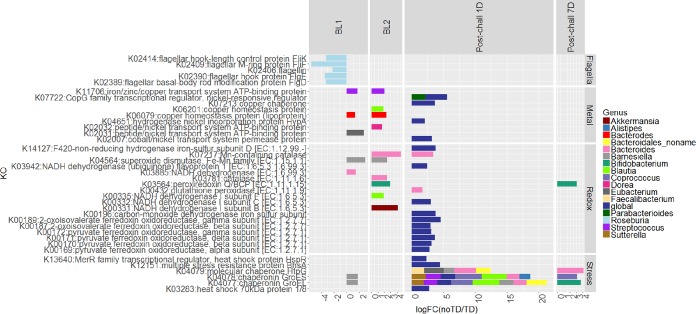
Significant differences in the gene expression profiles between no-TD and TD subjects at baseline (BL), 1 day postchallenge (Post-chall 1D), or 7 days postchallenge (Post-chall 7D).

Using KEGG orthologs (KOs), we evaluated differential expression between TD and no-TD subjects in the two baseline samples and in samples collected 1 day (D1) or 1 week (D7) after challenge with wild-type *S.* Typhi (Fig. 5). We observed that several enzymes and proteins associated with oxidative stress were expressed at higher levels in no-TD volunteers across all time points in this study. Genes encoding superoxide dismutase (K04564) in *Barnesiella* and catalase (K03781) and Mn-containing catalase (K07217) in *Bacteroides* were expressed at higher levels in no-TD volunteers at baseline. The latter enzyme, together with glutathione peroxidase (K00432) of *Bacteroides*, was also expressed at higher levels in no-TD volunteers 24 h postchallenge. Similarly, *Bifidobacterium* peroxiredoxin Q/BCP (K03564) was more abundant in samples from no-TD volunteers at baseline and 7 days postchallenge.

We also observed that genes encoding iron-sulfur binding enzymes (K00196 and K14127) were globally expressed at higher levels in no-TD volunteers at 24 h postchallenge (Fig. 5). NADH dehydrogenase, which carries crucial iron-sulfur center subunits for electron transfer, was overexpressed in no-TD volunteers at baseline in the genera *Akkermansia* (K00331), *Blautia* (K00335), and *Bacteroides* (K03885), as well as 24 h postchallenge globally (K00332 and K03942). Other genes encoding iron-sulfur-containing enzymes, including ferredoxin oxidoreductase using pyruvate (K00169 to K00172) and 2-oxoglutarate (K00187 and K00189) as the substrate, were globally overexpressed in no-TD volunteers at 24 h postchallenge.

Copper is another redox-active metal (18), and genes encoding copper homeostasis proteins displayed a higher level of expression in no-TD volunteers at baseline in *Bacteroides* (K06079) and *Blautia* (K06201), the iron/zinc/copper transport system ATD-binding protein (K11706) of *Streptococcus* was expressed at higher levels in no-TD volunteers at baseline, and the copper chaperone (K07213) was overexpressed globally in no-TD volunteers 24 h postchallenge. The Gene Ontology term copper ion binding (GO:0005507) also showed higher expression in no-TD volunteers at 24 h postchallenge, consistent with the KEGG analysis results.

In addition to redox-active metals, we found that genes encoding nickel-related proteins displayed a higher level of expression in no-TD volunteers at baseline and 24 h postchallenge. These include cobalt/nickel transport system permease protein (K02007, global), hydrogenase nickel incorporation protein (K04651, global), and CopG family transcriptional regulator, nickel-responsive regulator (K07722, global and in *Parabacteroides*), which were expressed at higher levels in no-TD volunteers 24 h postchallenge, as well as peptide/nickel transport system ATD-binding protein (K02031 in *Eubacterium* and K02032 in *Dorea*), which was more abundant in no-TD volunteers at baseline. In agreement with the above results, the GO terms nickel cation binding (GO:0016151) and divalent inorganic cation homeostasis (GO:0072507) were observed to be overrepresented in no-TD volunteers at 24 h postchallenge.

A gene encoding the heat shock protein HspR, which belongs to MerR transcriptional regulator (K13640), was expressed at higher levels in no-TD subjects at 24 h postchallenge. MerR family proteins have been known to activate the expression of genes required for metal efflux or detoxification, defense against oxidative stress, and drug resistance (18).

Comparison of the metatranscriptomic data sets from no-TD versus TD volunteers 24 h postchallenge revealed a higher level of expression of several stress response genes in no-TD volunteers, including the chaperonins GroEL (K04077) and GroES (K04078) from multiple genera, including *Blautia*, *Coprococcus*, *Bifidobacterium*, *Streptococcus*, *Sutterella*, *Barnesiella*, *Bacteroides*, and *Alistipes*. In addition, genes encoding the multiple stress resistance protein BhsA (K12151) and heat shock proteins (K03283 and 13640) were expressed at a higher level in no-TD volunteers at 24 h postchallenge. The GO terms chaperone binding (GO:0051087), unfolded protein binding (GO:0051082), and heat shock protein binding (GO:0031072) exhibited similar expression patterns as the KEGG profiles.

Flagellum-associated proteins from *Eubacterium* and *Roseburia* were overexpressed in TD volunteers at multiple times before challenge with *S.* Typhi. GO terms bacterial-type flagellum assembly (GO:0044780) and bacterial-type flagellum-dependent cell motility (GO:0071973) also displayed higher levels of expression in TD volunteers at baseline.

## DISCUSSION

One of the most important roles of the gut microbiota in health is to inhibit colonization and overgrowth of pathogenic species—a process called “colonization resistance.” This process involves a complex set of interactions among the constituents of the microbiota and between the microbiota and host (19, 20). Metatranscriptome analysis revealed two patterns of gene expression in the gut microbiota of our vaccine study participants that persisted from baseline through vaccination and challenge—the first was characterized by a high relative abundance of transcripts from the methanogen *Methanobrevibacter*, and the second was characterized by a diverse set of transcripts from representatives of the *Firmicutes* phylum. These two transcriptome community types were associated with different clinical outcomes following challenge with wild-type *S.* Typhi, i.e., the presence of *Methanobrevibacter* was associated with no TD, whereas the *Firmicutes* were associated with TD. One question raised by these results is whether, and how, the functional differences between the two transcriptome communities contributed to the different clinical outcomes that were observed.

Macrophages and lymphocytes are prominent in gut infiltrates following exposure to *S.* Typhi (21, 22), and macrophages are avid producers of reactive oxygen species (ROS) that exert bactericidal effects (23). The *Methanobrevibacter*-dominated community was associated with a lower risk of developing TD following challenge with wild-type *S.* Typhi. It is known that H_2_-utilizing methanogenic archaea in the human gut microbiota are extremely sensitive to free radicals, requiring a strongly reduced gut environment (redox potential < −200 mV) to survive (24, 25). It is possible that the highly reduced environment required for the survival of gut methanogens mitigates the effect of ROS produced following *Salmonella* infection, thereby altering the environmental signals that trigger a cascade of virulence factors necessary for host invasion (26). This notion was also supported by the observation that genes encoding multiple enzymatic antioxidants in several genera in the *Methanobrevibacter*-dominated community were highly expressed both at baseline and following challenge with wild-type *S.* Typhi in no-TD subjects. Our data suggest that multiple mechanisms may be employed to control the oxygen concentration in the *Methanobrevibacter* community and are consistent with recent reports that methanogenic archaea can be grown in the presence of oxygen when the culture medium is supplemented with the antioxidants ascorbic acid, uric acid, and glutathione (27, 28).

Oxidative stress can also disturb the homeostasis of redox-active metals, including iron, copper, cobalt, and nickel (18), which in turn can act as catalysts to further drive the production of ROS and reactive nitrogen species (RNS) (29). To maintain an optimal bioavailable concentration of a metal ion, cellular metabolism must be altered to balance metal uptake and efflux/storage processes. Several other observations from analysis of the metatranscriptome data suggest potential differences between the transcriptome community responses to oxidative stress. Subjects who did not develop typhoid also had a higher level of expression of genes encoding proteins containing iron-sulfur binding subunits as well as GO terms associated with divalent inorganic cation homeostasis and nickel/copper/molybdenum binding at baselines or postchallenge. The high expression of genes involved in metal homeostasis might reflect a response to the presumed oxidative stress induced by *S.* Typhi infection.

In order to invade, enteric pathogens must traverse both the inner and outer mucus layer that separates the gut lumen from the underlying epithelium. The relative abundance of *Akkermansia* spp., a clade of mucin-degrading bacteria, has been reported to correlate with the thickness of the mucus layer (30). It was therefore of interest to observe a positive correlation between *Methanobrevibacter* and *Akkermansia* in both the 16S rRNA gene and metatranscriptomic data sets, suggesting that potential differences in the mucus layer among subjects may also contribute to different clinical outcomes following challenge with wild-type *S.* Typhi.

Finally, gut methanogens have been reported to play a role in activation of the adaptive immune response by upregulating cell surface receptors CD86 and CD197 on monocyte-derived dendritic cells (moDCs) and expression of human β-defensin in moDCs exposed to Methanobrevibacter smithii *in vitro* (31). These findings suggest that methanogenic archaea may possess important immunomodulatory functions that could potentially impact outcomes following infection with enteric pathogens.

Analysis of the metatranscriptome data revealed that a suite of genes encoding proteins involved in flagellar biosynthesis and chemotaxis in the motile bacteria *Eubacterium* and *Roseburia* were consistently present at higher abundance in subjects who developed clinical symptoms following challenge with *S.* Typhi. It is possible that this observation reflects the fact that the diverse *Firmicutes* transcriptome community type is correlated with TD. On the other hand, bacterial flagellins, the major structural protein of flagella, and another flagellar hook protein, FlgE, have been reported to play an important role in mucosal immune activation of the Toll-like receptor 5 (TLR5) pathway (32–34). Our data are of interest in light of the findings of Cullender et al., who reported that antiflagellum antibodies immobilize commensal motile bacteria *in vitro* and also function to induce downregulation of flagellin gene expression by an unknown mechanism (35). The significantly greater abundance of flagellin genes in TD subjects is consistent with the possibility of an overall lower level of antiflagellin antibodies in the gut, suggesting possible differences in the innate immune response among the subjects in this study. While it is not possible to further speculate on the impact of differences in the overall level of commensal flagellin gene expression on host immune status and clinical outcome following exposure to wild-type *S.* Typhi, this relationship should be further examined in future studies.

To the best of our knowledge, this is the first demonstration that differences in the composition and function of the human gut microbiome correlate with the development of typhoid symptoms following exposure to wild-type *S.* Typhi. These observations have important implications in interpreting the efficacy of oral attenuated vaccines against enteric pathogens in diverse populations. The association between *Methanobrevibacter* and lack of disease symptoms is of interest, especially given that methanogens appear to represent a relatively minor fraction of the gut microbiota (36). Few studies have examined the contribution of methanogens to gut microbiota function. A previous study from our laboratory (16) examining the impact of a *Lactobacillus* probiotic on the function of the gut microbiota also described a transcriptome community dominated by *Methanobrevibacter* transcripts. Taken together, the data from these two studies are consistent with the notion that methanogens may be critically important to the overall function of the gut microbiota in a subset of individuals.

There is one important caveat to our study. The small intestine is the site of invasion for *S.* Typhi, from which it can disseminate to other tissues, including the liver, spleen, and gallbladder (37). Fecal samples are most representative of the ecosystem present in the colon; however, it is not practical to sample the microbiome of the small intestine in longitudinal human studies. Our data indicate that further exploration of the mechanisms by which differences in the gut microbiome can impact susceptibility to enteric pathogens in both humans and animal models may provide important new insights into the interplay between host, pathogen, and the microbiome.

## MATERIALS AND METHODS

### Subject recruitment and sample collection.

Longitudinal fecal samples were collected from healthy adults recruited in Oxford, United Kingdom (13). Volunteers received 3 doses of oral Ty21a (a licensed live attenuated typhoid vaccine, strain Ty21a), a single dose of M01ZH09 (an attenuated live typhoid vaccine candidate strain), or placebo, followed 28 days later by challenge with 10^4^ CFU of wild-type (wt) *S.* Typhi (Quailes strain), monitoring of clinical symptoms and hematological and biochemical responses, and daily microbiological sampling. Stool samples were collected at the screening interview (baseline 1), prior to the first vaccination visit (baseline 2), two time points during vaccination (days −28 and −26) for placebo and M01ZH09 groups but four time points during vaccination (days −32, −30, −28, and −26) for the Ty21a group, prior to challenge with *S.* Typhi (day 0), and five time points after challenge at day 0: 12 h, day 1, day 3, day 7, and day 10. Written informed consent was obtained, and all procedures were approved by National Research Ethics Service South Central—Oxford A (11/SC/0302). The clinical trial was sponsored and monitored by the Oxford University Clinical Trials and Research Governance Department. After collection, stool was mixed with RNAlater, homogenized, aliquoted into cryovials, and stored at −80°C until further processing.

### DNA extraction and 16S rRNA gene sequencing.

DNA was extracted from each of 283 stool specimens. For processing, samples were thawed at 4°C and, in aliquots of 0.15 g per tube, resuspended in 1 ml of 1× phosphate-buffered saline. Cell lysis was initiated with two enzymatic incubations, first, using 5 µl of lysozyme (10 mg ml^−1^; Amresco, Solon, OH), 13 µl of mutanolysin (11.7 U µl^−1^; Sigma-Aldrich), and 3 µl of lysostaphin (4.5 U µl^−1^; Sigma-Aldrich) for an incubation of 30 min at 37°C and, second, using 10 µl proteinase K (20 mg ml^−1^; Research Products International, Mt. Prospect, IL), 50 µl 10% SDS, and 2 µl RNase (10 mg ml^−1^) for an incubation of 45 min at 56°C. After the enzyme treatments, cells were disrupted by bead beating in tubes with lysing matrix B (0.1-mm silica spheres; MP Biomedicals, Solon, OH), at 6 m s^−1^ for 40 s at room temperature in a FastPrep-24 (MP Biomedicals). The resulting crude lysate was processed using the ZR fecal DNA miniprep kit (Zymo, Irvine, CA) according to the manufacturer’s recommendations. The samples were eluted with 100 µl of ultrapure water into separate tubes. DNA concentrations in the samples were measured using the Quant-iT PicoGreen double-stranded DNA (dsDNA) assay kit (Molecular Probes, Invitrogen, Carlsbad, CA).

### 16S rRNA gene sequence analysis.

Hypervariable regions V3 and V4 of the bacterial 16S rRNA gene were amplified with primers 319F and 806R as described previously (38). The libraries were sequenced on 250PE MiSeq runs. Quality trimming of these sequences was carried out using the procedures described previously (38) for 250-bp reads according to the following criteria: (i) reads were truncated upstream of >2 consecutive low-quality bases, (ii) no reads with ambiguous base calls were used, and (iii) reads with <150 bp after trimming were discarded. Demultiplexing was performed with QIIME (version 1.8.0) (39). UCHIME v5.1 was conducted for *de novo* chimera detection and removal. Sequences were clustered as operational taxonomic units (OTUs) based on a 97% cutoff with USEARCH. Taxonomic ranks were assigned to each sequence with the Ribosomal Database Project (RDP) Naive Bayesian Classifier v.2.2, using the Greengenes database of 16S rRNA gene sequences (August 2013) and a confidence value cutoff of 0.97.

### RNA extraction and sequencing.

Total RNA was extracted using a protocol described by Eloe-Fadrosh et al. (16). Briefly, cells from 250 µl homogenized stool were lysed using acid phenol, SDS, and aggressive bead beating using lysing matrix tubes (Qbiogene) and a FastPrep FP120 instrument (Qbiogene). Total RNA was extracted by three rounds of phenol-chloroform-isoamyl alcohol (IAA) extraction, ethanol precipitation, and DNase treatment using the Ambion Turbo DNA-free kit (Invitrogen catalog no. AM1907) followed by purification using a Qiagen RNeasy kit (catalog no. 74104) and quality control using an Agilent 2100 Expert Bioanalyzer. Contamination of genomic DNA was evaluated by 16S rRNA gene PCR. Subsequently, rRNA was depleted using a combined Gram-positive and Gram-negative Ribo-Zero rRNA removal kit (Epicentre Technologies) followed by a purification step using a Zymo RNA Clean and Concentrator kit (catalog no. R1015) and quality control using an Agilent RNA 6000 Nano kit (catalog no. 5067-1511). This protocol generally yields 150 to 300 ng from an initial input of 5 µg total RNA. Following a variation of the manufacturer’s protocol, the TruSeq RNA sample preparation kit (Illumina) was used to prepare RNA-seq libraries containing 6-bp indexes. Using AMPure XT beads (Beckman Coulter Genomics), cDNA was purified and library size selection was performed. Sequencing was performed using the Illumina HiSeq2000 platform with three samples multiplexed per lane. RNA extracted from the same samples as for RNA-seq was used to perform qRT-PCR (see Text S1 and Table S2 in the supplemental material).

10.1128/mBio.00686-18.1 TEXT S1  Quantitative reverse transcriptase PCR (qRT-PCR). Download TEXT S1, PDF file, 0.2 MB. Copyright © 2018 Zhang et al. 2018 Zhang et al. This content is distributed under the terms of the Creative Commons Attribution 4.0 International license.

### RNA-seq taxonomic identification and differential expression analysis.

Raw sequence data were processed with adaptive quality trimming and adapter removal using in-house quality control (QC) pipelines. rRNA was filtered out by aligning reads using Bowtie 2 (40) to rRNA reference sequence database SILVA Parc (41). The rRNA-filtered reads were analyzed with BMTagger (42) to identify putative human sequences that were then removed. The remaining reads were first used for taxonomical profiling by MetaPhlAn (15) followed by transcriptome profiling by HUMAnN2 (the HMP Unified Metabolic Analysis Network), in which nucleotide-level searches were conducted via Bowtie 2 against a functionally annotated pangenome database, ChocoPhlAn, and unmapped reads were searched on a translational level via Diamond (43). The resulting UniRef50 gene families were mapped to KEGG Orthogroups (KOs) and Gene Ontology (GO). Differential gene expression analysis was conducted with edgeR to study both global and genus-level gene expression patterns (44). The trimmed mean of M values (TMM) was utilized to estimate appropriate scaling factors for normalization among samples with the inherent assumption that the majority of counts are not differentially expressed (45).

### Clustering of taxonomic composition.

We removed any genus estimates less than 1% and 0.3% of sample sequences obtained from 16S rRNA gene sequencing and RNA sequencing, respectively, and then renormalized the remaining genus-level abundance estimates to sum to 1.0 for each sample. We next computed the mean and standard deviation (SD) of observed estimates for each predicted genus across all samples. Using these data, we mapped all remaining abundance estimates for each genus into a [0,1] range representing relative deviation from the genus’s mean abundance estimate in SD units. Our method involves Euclidean-space clustering, where the relevance of absolute values would, without this final normalization, force our method to focus only on the most-abundant genera when identifying similarity between samples. The clustering tool that we used as a core subroutine was cluster 3.0 (http://bonsai.hgc.jp/~mdehoon/software/cluster/software.htm). This tool performs *k*-means Euclidean-space clustering on a given set of vectors for a given value of *k*. We avoided the issue entirely by varying *k* between 3 and 20 inclusive and then ran cluster 1,000 times for each *k*.

### qRT-PCR.

The expression level of a subset of transcripts present in baseline samples was determined using qRT-PCR. DNA and RNA were copurified from the same stool samples used for RNA-seq using the MagAttract PowerMicrobiome DNA/RNA kit (Qiagen) using an automated liquid handling system. Total RNA was prepared from DNA/RNA through two rounds of DNase treatment using Turbo DNAfree enzyme (Ambion) according to the rigorous DNase treatment protocol. Complete digestion of DNA was confirmed by performing a 35-cycle endpoint PCR for the 16S rRNA gene, targeting the V3-V4 (nucleotide [nt ]318 to 806) amplicon region. Total RNA was converted to cDNA using SuperScript III reverse transcriptase according to the manufacturer’s protocol (Invitrogen) and with the use of random hexamer primers. The cDNA was diluted 1:20 into nuclease-free water (Ambion) before analysis using reverse transcription-quantitative PCR (qPCR). The qPCR was performed using SYBR green master mix (Life Technologies) with 10-µl reaction mixtures comprised of the following: 5 µl of 2× SYBR master mix, 1 µl of each of the 1 µM forward and reverse primers (Table S1), 1 µl of nuclease-free water (Ambion), and 2 µl of cDNA. Triplicate reactions were performed for each cDNA template and primer combination using a CFX 384 qPCR instrument (Bio-Rad). The qPCR conditions were as follows: 40 cycles of 95°C for 15 s and 60°C for 1 min, followed by a dissociation stage. The cycle threshold (*C*_*T*_) values were calculated using the Bio-Rad software. The *C*_*T*_ values of the biological replicates were averaged, and the standard deviation was calculated. The *C*_*T*_ values of target genes of each sample were normalized by subtracting from them the *C*_*T*_ value of the constitutively expressed RNA polymerase beta subunit, *rpoB*, resulting in the Δ*C*_*T*_ value of a particular gene for each sample.

### Accession number(s).

All subject data have been deposited in NCBI’s database of Genotypes and Phenotypes (dbGaP) under study accession no. phs001521.v1.p1 and are accessible to authorized users according to the NIH dbGAP system policies and procedures. Sequence reads from the 16S rRNA gene profiling and metatranscriptomic experiments are available at SRA under accession number PRJNA407499.

10.1128/mBio.00686-18.3 FIG S2  Quantitative reverse transcription-PCR (qPCR) analysis of select genes for baseline RNA samples from this study. The values represent the log RPKM (reads per kilobase per million) values from the RNA-seq result compared to –Δ*C*_*T*_ values from the qPCR result. Regression lines in blue are presented for each KO. Positive correlations between RNA-seq and qPCR were detected for all the tested KOs. Download FIG S2, PDF file, 0.2 MB. Copyright © 2018 Zhang et al. 2018 Zhang et al. This content is distributed under the terms of the Creative Commons Attribution 4.0 International license.
