# Supplementary material for: Compositional and Functional Differences in the Human Gut Microbiome Correlate with Clinical Outcome following Infection with Wild-Type Salmonella enterica Serovar Typhi
Source: mBio. 2018 May 8;9(3):e00686-18. doi: 10.1128/mBio.00686-18 (PMC5941076; doi:10.1128/mBio.00686-18)
Supplement: FIG S1 [file mbo002183863sf1.pdf]

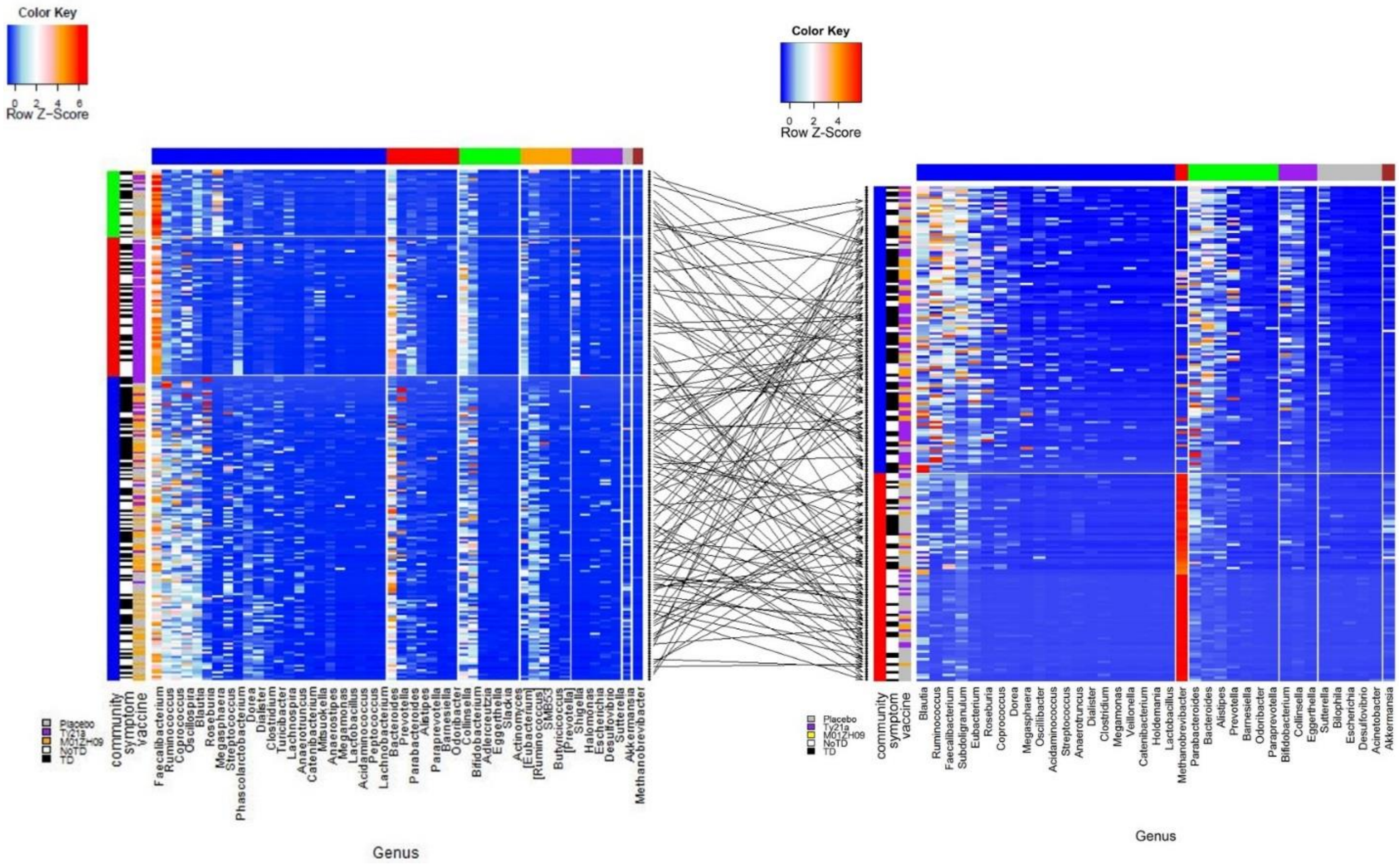

Supplemental Figure S1. Taxonomic abundance of the human gut microbiome estimated by 16S rRNA and metatranscriptome sequencing.

The two heat maps represent the same data presented in Figures 1 and 2. In the middle panel lines have been drawn to connect the same samples between the two heat maps.
