# Supplementary material for: Compositional and Functional Differences in the Human Gut Microbiome Correlate with Clinical Outcome following Infection with Wild-Type Salmonella enterica Serovar Typhi
Source: mBio. 2018 May 8;9(3):e00686-18. doi: 10.1128/mBio.00686-18 (PMC5941076; doi:10.1128/mBio.00686-18)
Supplement: TABLE S1 [file mbo002183863st1.pdf]

Supplemental Table S1. Bray-Curtis dissimilarity for each subject over time. Abbreviations: BL, baseline; Vacc, vaccination; PostVacc, post-vaccination; PreChall, pre-challenge; Post1D, 1-day post-challenge; Post7D, 7-day post-challenge.

Subject	outcome	vaccine	community	BL1-BL2	BL2-Vacc	Vacc-PostVacc	PostVacc-PreChall	PreChall-Post1D	Post1D-Post7D
568	NTD	Ty21a	Methano-dominated	0.100352	0.050331	0.09253405	0.06313452	0.07279914	NA
149	TD	Placebo	Methano-dominated	0.279608	0.295528	0.2731186	0.1733439	0.1332571	NA
250	TD	Ty21a	Diverse	0.298227	0.464459	0.3506802	0.3591618	0.5350303	0.2595225
552	TD	M01ZH09	Methano-dominated	0.156331	0.26152	0.6314366	0.7303599	0.6665661	0.2428583
586	NTD	M01ZH09	Diverse	0.296911	0.388285	0.3838152	0.2528591	0.3444417	0.2656658
777	TD	M01ZH09	Diverse	0.590884	0.711775	0.5148846	0.3413374	0.5810092	0.5453609
616	TD	Placebo	Methano-dominated	0.399438	0.675823	0.8324804	0.3314456	0.5097165	NA
926	NTD	Placebo	Methano-dominated	0.322587	0.837017	0.668666	NA	NA	NA
783	TD	Placebo	Methano-dominated	0.317479	0.537594	0.5312001	0.6655572	NA	NA
2	TD	Ty21a	Diverse	0.222468	0.202786	0.1761187	0.2686047	0.117927	0.3026768
703	TD	Ty21a	Diverse	0.201919	0.341993	0.2271009	0.2930581	0.1996614	NA
745	NTD	M01ZH09	Methano-dominated	0.504939	0.370014	0.42202045	0.22889498	0.04074566	0.17565265
373	TD	Placebo	Diverse	NA	NA	0.4673189	0.5269238	NA	NA
857	NTD	Ty21a	Diverse	0.407542	0.312598	0.2636531	0.1995709	0.4120577	0.5818702
644	TD	Ty21a	Diverse	0.237509	NA	NA	0.2727735	0.1664675	NA
859	NTD	Placebo	Diverse	NA	NA	NA	NA	0.3321297	0.4595007
467	TD	M01ZH09	Diverse	0.487953	0.512243	0.8133012	0.5449538	0.4202522	0.2888658
403	NTD	Ty21a	Diverse	0.513311	0.417696	0.3479852	0.5573594	0.5563995	0.5488997
968	TD	Ty21a	Diverse	0.261966	0.407359	0.2194428	0.1522348	0.1951248	0.2653347
87	TD	Placebo	Diverse	0.454472	0.620075	0.727897	0.6692778	0.1275654	0.1354279
317	TD	M01ZH09	Diverse	0.48361	0.519546	0.4738748	NA	NA	0.3477299
330	NTD	Placebo	Diverse	0.269879	0.239558	0.3225024	0.3970274	NA	NA
672	NTD	Placebo	Methano-dominated	0.303945	0.16703	0.05572576	0.24312717	0.15739419	0.41581142
882	TD	M01ZH09	Diverse	0.318344	0.300868	0.2115798	0.2583248	0.2412918	NA
765	NTD	Ty21a	Diverse	0.148388	0.436634	0.3335784	0.5618702	0.1388239	NA
642	NTD	Ty21a	Methano-dominated	0.082587	0.366503	0.40611002	0.18018878	0.205705	0.12230673
781	NTD	Placebo	Methano-dominated	0.049947	0.128086	0.1699083	0.08815929	0.07999262	NA
