# Supplementary material for: Compositional and Functional Differences in the Human Gut Microbiome Correlate with Clinical Outcome following Infection with Wild-Type Salmonella enterica Serovar Typhi
Source: mBio. 2018 May 8;9(3):e00686-18. doi: 10.1128/mBio.00686-18 (PMC5941076; doi:10.1128/mBio.00686-18)
Supplement: TABLE S2 [file mbo002183863st2.pdf]

Supplemental Table S2. Primers used in this study

Gene	Predicted Protein Function	Taxa	Primer ID	Sequence 5'-3'	Source
K02390	flagellar hook protein FlgE	*Roseburia*	Roseburia_UniRef50_G2T3E1_F	TGGTAACAATATCGCCAACG	This study
			Roseburia_UniRef50_G2T3E1_R	ACCGGTCGTAACACCAAGAC	This study
K02406	flagellin	*Roseburia intestinalis*	Roseburia_intestinalis_D4L1N7_F	GCATCGATGGAACGGCTTTC	This study
			Roseburia_intestinalis_D4L1N7_R	TTTCACTCAGCGCACCATCT	This study
		Roseburia intestinalis	Roseburia_intestinalis_P21991andQ39YI9_F	GCGCACAGGCAAAATCTTCA	This study
			Roseburia_intestinalis_P21991andQ39YI9_R	AATCCACGGATCTGGCTTCT	This study
		Roseburia hominis	Roseburia_hominis_G2T5W2_F	ATTGGATGACGTGGCGAACT	This study
			Roseburia_hominis_G2T5W2_R	CCGGCAGAATCTGTCTTGGT	This study
K06933	uncharacterized protein	*Methanobrevibacter smithii*	M_smithi_A5UJN4_F	TGCGAAGACTGTGGTTTTCA	This study
			M_smithi_A5UJN4_R	CAGCTGCAGGTGTCATCTGA	This study
K14081	methanol corrinoid protein	*Methanobrevibacter smithii*	M_smithi_UniRef50_Q2NHM9_F	TGTGGTGGAGGAGCTGTAAG	This study
			M_smithi_UniRef50_Q2NHM9_R	CGGTACGAATTCCCCTACAA	This study
rpoB	RNA polymerase, beta subunit	*Roseburia hominis*	Rhominis_rpoB_F	CCGGTGTTGTTGAGAGCTCT	This study
			Rhominis_rpoB_R	AACACGATCGGCTTCTGGTT	This study
rpoB	RNA polymerase, beta subunit	*Roseburia intestinalis*	Rintestinalis_rpoB_F	TGTTCCTGGTGTCGGTGATG	This study
			Rintestinalis_rpoB_R	TCTCACCGAAAATCGCACGA	This study
rpoB	RNA polymerase, beta subunit	*Methanobrevibacter smithii*	Ms_rpoB_F	AAGGGATTTGCACCCAACAC	(1)
			Ms_rpoB_R	GACCACAGTTAGGACCCTCTGG	(1)

Reference

1. Dridi B, Henry M, El Khechine A, Raoult D, Drancourt M. 2009. High prevalence of Methanobrevibacter smithii and Methanosphaera stadtmanae detected in the human gut using an improved DNA detection protocol. PloS one 4:e7063.
