# Supplementary material for: Compositional and Functional Differences in the Human Gut Microbiome Correlate with Clinical Outcome following Infection with Wild-Type Salmonella enterica Serovar Typhi
Source: mBio. 2018 May 8;9(3):e00686-18. doi: 10.1128/mBio.00686-18 (PMC5941076; doi:10.1128/mBio.00686-18)
Supplement: TEXT S1 [file mbo002183863s1.pdf]

Supplemental Method S1.

Quantitative Reverse Transcriptase PCR (qRT-PCR)

The gene expression levels in baseline samples for a subset of genes that were found to be differentially expressed between the two transcriptome communities were determined using qRT-PCR. DNA and RNA were co-purified from the same stool samples used for RNA-Seq using the MagAttract PowerMicrobiome DNA/RNA kit (Qiagen) using an automated liquid handling system. Total RNA was prepared from DNA/RNA through two rounds of DNAse treatment using Turbo DNAfree enzyme (Ambion) according to the rigorous DNAse treatment protocol. Complete digestion of DNA was confirmed by performing a 35-cycle endpoint PCR reaction for 16S rRNA, targeting the V3V4 (318-806 nt) amplicon region. Total RNA was converted to cDNA using SuperScript III reverse transcriptase according to manufacturer's protocol (Invitrogen) and with the use of random hexamer primers. The cDNA was diluted 1:20 into nuclease free water (Ambion) before analysis using qPCR. The qPCR was performed using SYBR Green master mix (Life Technologies) with 10 µl reactions comprised of the following: 5 µl of 2X SYBR master mix, 1 µl of each of the 1 µM forward and reverse primers (Supplemental Table 1), 1 µl of nuclease free water (Ambion), and 2 µl of cDNA. Triplicate reactions were performed for each cDNA template and primer combination using a CFX 384 qPCR instrument (BIORAD). The qPCR conditions were as follows: 40 cycles of 95°C for 15 s and 60°C for 1 min, followed by a dissociation stage. The cycle threshold (Ct) values were calculated using the BIORAD software. The Ct values of the biological replicates were averaged and the standard deviation was calculated. The Ct values of target genes of each sample were normalized by subtracting from it the Ct value of the constitutively-expressed RNA polymerase beta subunit, *rpoB*, resulting in the ΔCt value of a particular gene for each sample.
