# Supplementary material for: Compositional and Functional Differences in the Human Gut Microbiome Correlate with Clinical Outcome following Infection with Wild-Type Salmonella enterica Serovar Typhi
Source: mBio. 2018 May 8;9(3):e00686-18. doi: 10.1128/mBio.00686-18 (PMC5941076; doi:10.1128/mBio.00686-18)
Supplement: FIG S2 [file mbo002183863sf2.pdf]

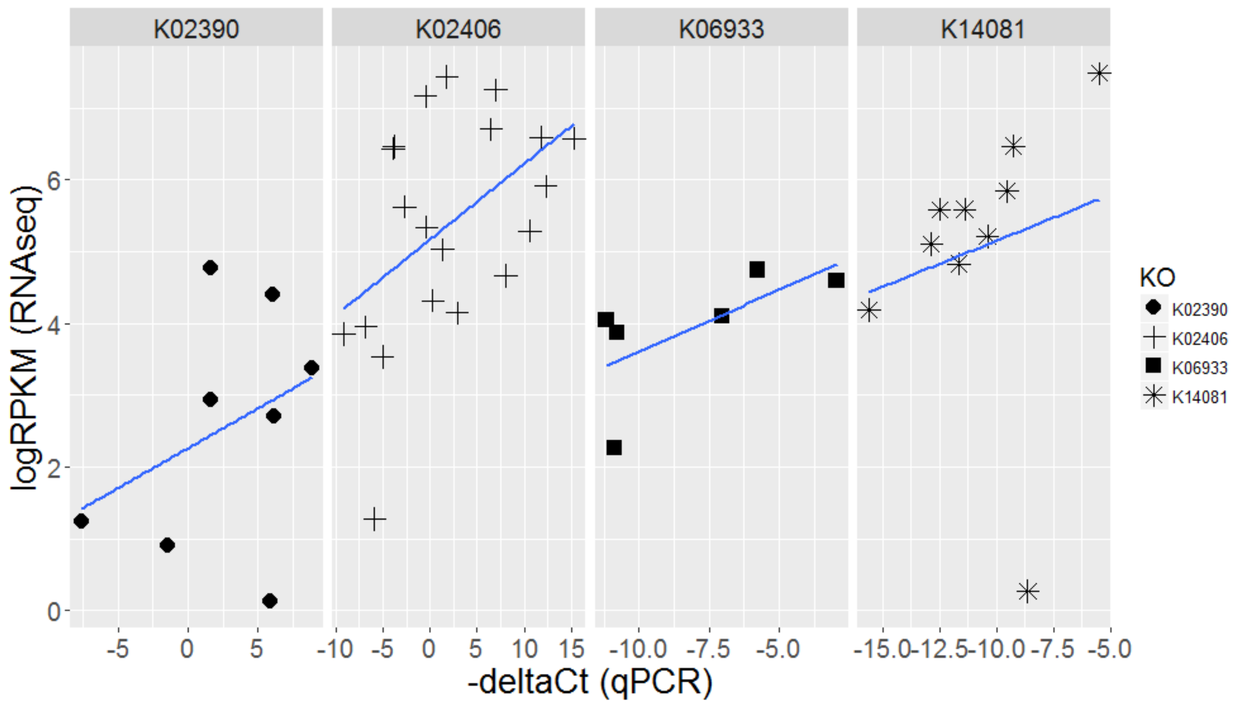

Supplemental Figure S2. Quantitative reverse transcription PCR (qPCR) analysis of select genes for baseline RNA samples from this study. The values represent the logRPKM values from the RNA-Seq result compared to –ΔCt values from the qPCR result. Regression lines in blue are presented for each KO. Positive correlations between RNAseq and qPCR were detected for all the tested KOs.
